# Epidemiological Comparison of Anorectal Malformation With Other Gastrointestinal Abnormalities in Patients in the Pediatric Ward

**DOI:** 10.7759/cureus.23136

**Published:** 2022-03-14

**Authors:** Sajeel Saeed, Abdul Rauf Khalid, Muhammad Farhan, Jawad Basit, Kashif Tousif, Tehseen Haider, Noor Us Sabah, Mudassar Fiaz Gondal, Mohammad Ebad ur Rehman

**Affiliations:** 1 Department of Surgery, Holy Family Hospital, Rawalpindi, PAK; 2 Department of Internal Medicine, Holy Family Hospital, Rawalpindi, PAK; 3 Department of Pediatric Surgery, Holy Family Hospital, Rawalpindi, PAK

**Keywords:** imperforate anus, gastroenterology, pediatrics, epidemiology, congenital anomalies, prevalence, anorectal malformation

## Abstract

Background

Anorectal malformations are congenital defects wherein there is defective development of the anus and rectum. For babies born with congenital anorectal malformations, prompt treatment is crucial which requires detection of the anomalies at the earliest. This study aimed to determine the epidemiology ofanorectal malformations in the Pediatric Unit of a tertiary care hospital in Pakistan over a period of 19 months.

Methodology

An analytical cross-sectional study was conducted retrospectively from January 2020 to September 2021 using a non-randomized consecutive sampling technique. Patients aged less than eight years were included, whereas burnt, torn, and incomplete records from the Hospital Management Information System (HMIS) were excluded. SPSS version 26 (IBM Corp., Armonk, NY, USA) was used for data entry and analysis. Binomial and multinomial logistic regression were applied for analyzing the association between explanatory and dependent variables.

Results

Of the 1,108 patients, 72 (6.5%) patients had anorectal malformations. Gastrointestinal diseases made up about 64.3% of all diseases. Among gastrointestinal causes, the prevalence of anorectal malformation was up to 10.1%. The mortality of anorectal malformation patients was low (2.85%) compared to mortalities due to other gastrointestinal abnormalities (8.25%). Anorectal malformation had significantly lower odds of mortality (adjusted odds ratio = 0.19, p < 0.05) compared to other gastrointestinal abnormalities.

Conclusions

This study has provided data about the prevalence of anorectal malformation and its mortality which were calculated as 6.5% and 2.58%, respectively. Female gender, neonates, and delayed presentation were seen to have higher mortality, highlighting the need to screen all neonates pre- and post-natally to avoid any misdiagnosis.

## Introduction

According to the National Institutes of Health, US Department of Health and Human Services, about one in every 5,000 neonates is born with anorectal malformations, with a slightly increased preponderance among males [1]. Anorectal malformations are congenital defects wherein there is defective development of the anus and rectum. Although the term seemingly refers to the child’s outward appearance, it often contradicts the true complexity and magnitude of the malformation occurring beneath. Anorectal deformities constitute a wide range of congenital defects in the terminal portions of the gut and urogenital tracts. It can either be isolated, in which the disease is confined to the rectum and anus, or it may be associated with other additional congenital anomalies of various systems of the body. According to a study based on a geographically defined population, 49.4% of neonates with anorectal malformations had other associated malformations. Malformations of the urogenital system (81.1%) and the skeletal system (45.5%) were the most predominant malformations occurring with anorectal malformations, followed by abnormalities of the cardiovascular, digestive, and central nervous systems [2]. If not diagnosed at birth, anorectal malformations usually present later in life with defective bowel movements. Long-term management of bowel-related chronic symptoms and surgery depending on the type and severity of anorectal malformation remain the main treatment modalities. Early recognition and management are recommended in the treatment of children with anorectal malformations to prevent sepsis and other morbidities related to intestinal obstruction [3].

The exact cause of anorectal malformations remains unknown. Currently, the Krickenbeck classification model of anorectal malformation is followed which is based on specific malformations with therapeutic and prognostic consequences. A population-based study conducted in Italy found vestibular and perineal fistulas to be the most frequently occurring anorectal malformations in females and perineal and rectourethral fistulas to be the most occurring anorectal malformation in males [4]. The same study found the 20-year survival probability of patients affected by anorectal malformation to be 86.7%, with most cases of deaths occurring during the first months of life; one-month, three-month, and 12-month survival probabilities were found to be 92.5%, 90.9%, and 89.7%, respectively, and no significant differences were observed according to gender or the type of anorectal malformation [3]. Pakistan has a large load of unmet surgical needs in neonates, and similar to other developing countries, local pediatricians face challenging and complex clinical cases.

This study aimed to determine the epidemiology and assess the survival rate of anorectal malformations in babies admitted to the Pediatric Unit of Holy Family Hospital, Pakistan, over a period of two years (2020-2021). The prevalence, clinical manifestation, and survival rate of anorectal malformation in Pakistan may be considerably different from other regions of the world. The exact magnitude of occurrence will help experts adopt necessary measures and interventions to provide the best possible quality of life based on the associated conditions and types of anorectal malformation.

## Materials and methods

An analytical cross-sectional study was conducted in the Pediatric Surgery Department of a tertiary care hospital over 19 months. The study data were collected retrospectively from January 2020 to September 2021 using a non-randomized consecutive sampling approach. Cases were identified through the Hospital Management Information System (HMIS). The medical record files of these cases were obtained from the Data Maintenance room of the Pediatric Surgery Department of the hospital. This study included all patients with a confirmed diagnosis of anorectal malformation aged less than eight years who presented within the study duration. The exclusion criteria included medical record files with incomplete demographic details, incomplete labs, missing imaging results, missing clinical examination notes, and torn and burnt files. Patients having anorectal malformation as part of multiple congenital anomalies (MCAs) and those who were diagnosed with anorectal malformation but underwent surgical interventions such as colostomy, anorectoplasty, and reversal of colostomy at a different hospital were also excluded from the study. Neonates were diagnosed as having anorectal malformation by first clinical examination after birth. Others presented in the Surgical Outpatient Department (OPD) with symptoms of intestinal obstruction. A few presented with intestinal obstruction and septic shock to the surgical Emergency Room (ER). The diagnosis involved clinical examination by an expert pediatric surgeon followed by ultrasonography. Magnetic Resonance Imaging (MRI) was done in selected patients. Out of the total 1,293 patients who presented in the pediatric ward, 185 patients were excluded from the study after applying the exclusion criteria. Data from 1,108 cases were finally entered in SPSS version 26 (IBM Corp., Armonk, NY, USA) and analyzed.

Demographic variables were taken from the medical record files. The age of the patient along with sex, diagnosis, system, outcome, mode of admission, date of admission, and date of discharge were obtained for each patient. Ethical approval was taken from the Institutional Research Forum of Rawalpindi Medical University. Consent for data collection was taken from the head of the Department of Pediatric Surgery. The need for individual consent was waived off by the Institutional Research Forum of Rawalpindi Medical University. The data collection procedure was in compliance with institutional and national ethical guidelines, as well as the most recent version of the Helsinki Declaration. The manuscript follows Strengthening The Reporting of Observational Studies in Epidemiology (STROBE) guidelines for reporting a cross-sectional study.

Data were entered and analyzed using SPSS version 26. Descriptive statistics included mean, standard deviation, and frequencies for quantitative variables, whereas percentages and proportions were computed for qualitative variables. The normality of the data was checked using Kolmogorov-Smirnov and Shapiro-Wilk tests. Pearson chi-square and Fisher’s exact tests were also applied. Binomial and multinomial logistic regression were applied to check the association between dependent and independent variables. Graphs were prepared using Microsoft Excel.

## Results

Out of 1,108 patients, 766 (69.1%) were males and 342 (30.9%) were females. The age of the patients was divided into three groups: neonate (<28 days), 28 days to two years, and more than two years. A total of 119 (10.7%) patients were neonates, 288 (26.0%) patients in the 28 days to two years age group, and 701 (63.3%) patients in more than two years age group. A total of 578 (52.2%) patients presented in the year 2020, whereas 530 (47.8%) patients presented in 2021. Most patients presented in the pediatric ward were due to gastrointestinal problems (64.3%). A total of 45% of patients presented through the OPD, 46.4% through the ER, 5.6% through the neonatal intensive care unit (NICU), and 3.0% through medical operation theater (M-OT).

The prevalence of anorectal malformation was 6.5%. Solely out of gastrointestinal causes, the prevalence of anorectal malformation was 10.1%. Using binomial and multinomial logistic regression, females had higher odds of having anorectal malformation (adjusted odds ratio (AOR) = 1.33, 95% confidence interval (CI) = 0.78, 2.16) compared to males. Taking the two to eight-year age group as the reference category, <28-day age group (AOR = 4.82, 95%CI: 2.46, 9.37) and 28 days to two-year age group (AOR = 1.37 95% CI = 0.76, 2.45) had higher odds of anorectal malformation. The association of anorectal malformation with different demographic indicators is presented in Table 1.

**Table 1:** Prevalence and association of demographic variables with anorectal malformation. *Significance in logistic regression with p-values less than 0.05; **significance in logistic regression with p-values less than 0.001. COR = crude’s odds ratio; AOR = adjusted odds ratio; CI = confidence interval; OPD = outpatient department; ER = emergency room; NICU = neonatal intensive care unit; M-OT = medical operation theater

Variables	Anorectal malformation	Other causes	COR (95% CI)	AOR (95% CI)
	n (%)	n (%)		
Gender				
Male	46 (6.0%)	720 (94.0%)	1	1
Female	26 (7.6%)	316 (92.4%)	1.28 (0.78, 2.12)	1.33 (0.78, 2.16)
Age groups				
<28 days	18 (15.1%)	101 (84.9%)	3.39 (1.85, 6.21)**	4.82 (2.46, 9.37)**
28 days to 2 years	19 (6.6%)	269 (93.4%)	1.34 (0.75, 2.39)	1.37 (0.76, 2.45)
2 years to 8 years	35 (5.0%)	666 (95.0%)	1	1
Year				
2020	33 (5.7%)	545 (94.3%)	1	1
2021	39 (7.4%)	491 (92.6%)	1.31 (0.81, 2.12)	1.15 (0.69, 1.88)
Mode of admission				
OPD	51 (10.2%)	448 (89.8%)	3.41 (0.81, 14.39)	6.71 (1.51, 29.92)*
ER	17 (3.3%)	497 (96.7%)	1.03 (0.23, 4.55)	1.56 (0.34, 7.17)
NICU	2 (3.2%)	60 (96.8%)	1	1
M-OT	2 (6.1%)	31 (93.5%)	1.93 (0.26, 14.41)	2.38 (0.31, 18.49)

**Figure 1:**
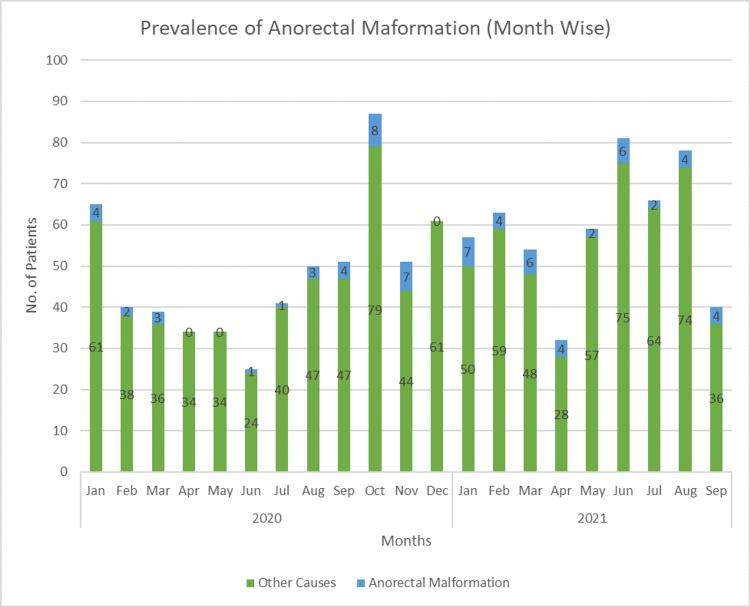
Month-wise prevalence of anorectal malformation.

Figure 1 shows the month-wise number of cases of anorectal malformation. The highest number of patients (85) was admitted in October 2020, and the highest number of cases of anorectal malformation was also reported in October 2020. No patient with a case of an imperforate anus or anorectal malformation was admitted in the months of April, May, and December 2020.

Using Pearson’s chi-square test, no significant difference in mortality was found in gender for anorectal malformation (χ^2^ = 1.16, p = 0.28), whereas a significant difference between males and females was found for patients who presented with other diseases (χ^2^ = 6.30, p = 0.01). For age groups, significant differences in mortalities were found for both patients including anorectal malformation (χ^2^ = 6.17, p = 0.04) and patients excluding anorectal malformation (χ^2^ = 88.36, p = 0.00). Table 2 shows the association of demographic indicators with mortality.

**Table 2:** Demographic comparison of mortalities of anorectal malformation patients and other diseases. *Significance in Pearson’s chi-square test with p-values less than 0.05; **significance in Pearson’s chi-square test with p-values less than 0.001. OPD = outpatient department; ER = emergency room; NICU = neonatal intensive care unit; M-OT = medical operation theater

Variables	Outcome (in anorectal malformation)		Chi-square, p-value	Outcome (excluding anorectal malformation)		Chi-square, p-value
	Expired	Discharge		Expired	Discharge	
Gender			χ^2^ = 1.16, p = 0.28			χ^2^ = 6.30, p = 0.01
Male	2 (4.3%)	44 (95.7%)		45 (6.2%)	675 (93.8%)	
Female	0 (0.0%)	26 (100%)		34 (10.8%)	282 (89.2%)	
Age groups			χ^2^ = 6.17, p = 0.04			χ^2^ = 88.36, p = 0.00
<28 days	2 (11.1%)	16 (88.9%)		31 (30.7%)	70 (69.3%)	
28 days to 2 years	0 (0.0%)	19 (100%)		21 (7.8%)	24 (92.2%)	
2 years to 8 years	0 (0.0%)	35 (100%)		27 (4.1%)	639 (95.9%)	
Year			χ^2^ = 1.74, p = 0.18			χ^2^ = 11.65, p = 0.01
2020	0 (0.0%)	33 (100%)		27 (5.0%)	518 (95.0%)	
2021	2 (5.1%)	37 (94.9%)		52 (10.6%)	439 (89.4%)	
Mode of admission			χ^2^ = 6.65, p = 0.08			χ^2^ = 24.27, p = 0.00
OPD	0 (0.0%)	51 (100%)		20 (4.5%)	428 (95.5%)	
ER	2 (11.8%)	15 (88.2%)		44 (8.9%)	453 (91.1%)	
NICU	0 (0.0%)	2 (100%)		13 (21.7%)	47 (78.3%)	
M-OT	0 (0.0%)	2 (100%)		2 (6.5%)	29 (93.5%)	

Using logistic regression, females had higher odds of mortality (AOR = 1.56, 95% CI = 0.96, 2.54) compared to males among all patients. Neonates (AOR = 8.69, 95% CI = 4.77, 15.82) and less than two-year-old babies (AOR = 1.83, 95% CI = 1.01, 3.32) had significantly higher odds of expiry compared to babies aged more than two years. Taking other diseases as the reference category, anorectal malformation or imperforate anus had significantly fewer odds of mortality (AOR = 0.19, 95% CI = 0.04, 0.82). Compared to OPD, patients coming from the ER (AOR = 1.70, 95% CI = 0.96, 3.03) and NICU (AOR = 2.75, 95% CI = 1.17, 6.43) into the pediatric ward had more odds of expiry. Table 3 shows the association of demographic variables with mortalities of anorectal malformation patients.

**Table 3:** Association of demographic variables with mortality index of anorectal malformation *Significance in logistic regression with p-values less than 0.05; **significance in logistic regression with p-values less than 0.001. COR = crude’s odds ratio; AOR = adjusted odds ratio; CI = confidence interval; OPD = outpatient department; ER = emergency room; NICU = neonatal intensive care unit; M-OT = medical operation theater

Variables	Outcome		COR (95% CI)	AOR (95% CI)
	Expired	Discharge		
Gender				
Male	47	719	1	1
Female	34	308	1.68 (1.06, 2.67)*	1.56 (0.96, 2.54)
Age groups				
<28 days	33	86	9.57 (5.49, 16.70)**	8.69 (4.77, 15.82)**
28 days to 2 years	21	267	1.96 (1.09, 3.53)*	1.83 (1.01, 3.32)*
2 years to 8 years	27	674	1	1
Year				
2020	27	551	1	1
2021	54	476	2.31 (1.43, 3.73)*	1.85 (1.12, 3.05)*
Diagnosis				
Anorectal malformation	2	70	0.34 (0.08, 1.43)	0.19 (0.04, 0.82)*
Other diagnoses	79	957	1	1
System				
Gastrointestinal system	53	659	1.06 (0.66, 1.70)	1.04 (0.63, 1.72)
Other	28	368	1	1
Mode of admission				
OPD	20	479	1	1
ER	46	468	2.35 (1.37, 4.04)*	1.70 (0.96, 3.03)
NICU	13	49	6.35 (2.97, 13.55)**	2.75 (1.17, 6.43)*
M-OT	2	31	1.54 (0.34, 6.91)	0.84 (0.17, 3.97)

## Discussion

Anorectal malformation puts a huge burden on global pediatric surgery and constitutes different variations of patterns from simple skin-level defects to more complex cases involving various urogenital and anorectal portions [5]. Anorectal malformations can occur as isolated defects or they can present as part of a syndrome. It can be further classified into two types of lesions: low-lying and high-lying. In clinical practice, these subtypes significantly affect the prognosis. The etiology of anorectal malformation is thought to be multifactorial and therefore both genetics and environmental factors, such as tobacco, caffeine, and alcohol, are likely involved in its development [6]. On the contrary, folic acid supplementation has been reported to reduce the risk of occurrence of anorectal malformations [7,8]. These lesions can be diagnosed prenatally by MRI, ultrasonography, and examination at the time of birth [9]. Posterior sagittal anoplasty and colostomy are preferred temporary surgical measures at birth for low-lying and high-lying lesions, respectively [10]. These defects significantly affect the quality of life of an individual and put severe distress on parents and carers alike.

We conducted this study to determine the prevalence of anorectal malformations in patients presenting at the Pediatric Unit of Holy Family Hospital, Rawalpindi. In this study, the prevalence of anorectal malformation was found to be 6.5%, while a study from Uganda and Tanzania reported the prevalence to be 24.7% and 19.6%, respectively [11,12]. This low prevalence was likely because of underdiagnosis or misdiagnosis. In our study, females had a higher odds ratio of having anorectal malformation in contrast to many studies that indicate male predominance; however, many similar studies have pointed to an equal distribution [5,13]. Males are reported to have been affected more when the severity of anorectal malformation is taken into consideration [14]. No significant difference in mortality was found in gender for anorectal malformation, which is consistent with a similar study conducted in Kenya [15]. Neonates are reported to have higher mortality compared to other age groups, as was found in our study. This is consistent with the findings of a population-based study conducted in Italy while contrary to the study conducted in Kenya [4,15].

Most patients report to the emergency department with complaints of intestinal obstruction and sepsis, both of which can lower the prognosis and prove fatal. As in our study, patients who present through the ER with a delayed diagnosis have a higher odds ratio of mortality, which is consistent with other studies that support higher mortality in cases of delayed diagnosis [16]. Anorectal malformations are also associated with multiple congenital anomalies and VACTERL (vertebral defects, anal atresia, cardiac defects, tracheoesophageal fistula, renal anomalies, and limb abnormalities) with incidences ranging from 45% to 75% causing high mortality and morbidity [17]. Diagnosis in such cases may be delayed due to several factors, such as lack of access to tertiary care facilities, lack of imaging modalities, childbirth outside of a healthcare facility, or improper examination of the neonate at the time of birth by the medical practitioner.

The mortality rate in our study was 2.1% compared to the Western world where it is reported to be 6% [18]. It may range from as low as 0.1-11% [3], but can be as high as 31%, as reported by a study conducted in Jaipur, India, and Kenya [10,15]. These differences in mortality rates may occur because of variations in healthcare resources, road infrastructure which leads to delayed presentation, low access to advanced equipment and facilities, improper documentation, and lack of expertise of medical practitioners, especially in the developing world where there is a shortage of expert pediatric surgeons. This lack of expertise can cause complications pre- and post-operatively which significantly increases mortality and morbidity.

This is the first study that discussed anorectal malformation and its prevalence in a larger sample size in our geographical area. There are many limitations to our study. First, because this is a cross-sectional study, this study cannot infer the cause-effect relationship. Second, the data collection was done using a non-randomized sampling approach, so the results cannot be generalized to the entire population. Third, as the prevalence was only calculated in the specific population (hospital) and not in the whole population, Berkson’s bias can occur. Fourth, as the data collection was done through past medical and surgical records in a single center, it is advisable to perform data collection using a multicentered approach.

## Conclusions

Anorectal malformation is a common congenital disease that severely affects the quality of life of children, and the lack of experienced pediatric surgeons, especially in the developing world, adds to it. This study has provided data regarding the prevalence of anorectal malformation, which was reported to be 6.5%. Mortality in this study was calculated to be 2.5% which is low compared to other studies. Mortality was associated with both the age of the patient, with high mortality among neonates, and delayed presentation with complications through ER. Data from other hospitals in the country should be gathered to find the national prevalence of anorectal malformations. Further, all neonates at birth should be properly screened even prenatally to avoid misdiagnosis which can result in delayed presentation and high mortality.
